# Supplementary material for: Electromagnetic-guided versus endoscopic placement of nasoenteral feeding tubes: protocol for a systematic review and meta-analysis
Source: BMJ Open. 2021 Mar 10;11(3):e044637. doi: 10.1136/bmjopen-2020-044637 (PMC7949433; doi:10.1136/bmjopen-2020-044637)
Supplement: Supplementary data [file bmjopen-2020-044637supp001.pdf]

Online supplementary appendix 1. Search strategies

MEDLINE (PubMed format)

up to 30 September 2020.

1. electromagn* [tiab] OR cortrak [tiab]
2. Endoscopy [mesh] OR endoscop* [tiab]
3. Enteral Nutrition [mesh] OR nasoenteral [tiab] OR nasojejunal [tiab] OR nasoduodenal [tiab] OR postpyloric [tiab] OR post-pyloric [tiab] OR nasointestinal [tiab] OR nasoenteric [tiab]
4. randomized controlled trial [pt] OR controlled clinical trial [pt] OR randomized [tiab] OR placebo [tiab] OR clinical trials as topic [mesh: noexp] OR randomly [tiab] OR trial [ti]
5. animals [mesh] NOT humans [mesh]
6. 4 NOT 5
7. 1 AND 2 AND 3 AND 6

EMBASE (Ovid format)

up to 30 September 2020.

1. Randomized controlled trial/
2. Controlled clinical study/
3. random$.ti,ab.
4. randomization/
5. intermethod comparison/

6. placebo.ti,ab.

7. (compare or compared or comparison).ti.

8. ((evaluated or evaluate or evaluating or assessed or assess) and (compare or compared or comparing or comparison)).ab.

9. (open adj label).ti,ab.

10. ((double or single or doubly or singly) adj (blind or blinded or blindly)).ti,ab.

11. double blind procedure/

12. parallel group$1.ti,ab.

13. (crossover or cross over).ti,ab.

14. ((assign$ or match or matched or allocation) adj5 (alternate or group$1 or intervention$1 or patient$1 or subject$1 or participant$1)).ti,ab.

15. (assigned or allocated).ti,ab.

16. (controlled adj7 (study or design or trial)).ti,ab.

17. (volunteer or volunteers).ti,ab.

18. human experiment/

19. trial.ti.

20. or/1-19

21. random$ adj sampl$ adj7 (“cross section$” or questionnaire$1 or survey$ or database$1)).ti,ab. not (comparative study/ or controlled study/ or randomi?ed controlled.ti,ab. or randomly assigned.ti,ab.)

22. Cross-sectional study/ not (randomized controlled trial/ or controlled clinical study/ or controlled study/ or randomi?ed controlled.ti,ab. or control group$1.ti,ab.)

23. (((case adj control$) and random$) not randomi?ed controlled).ti,ab.

24. (Systematic review not (trial or study)).ti.

25. (nonrandom$ not random$).ti,ab.

26. “Random field$”.ti,ab.

27. (random cluster adj3 sampl$).ti,ab.

28. (review.ab. and review.pt.) not trial.ti.

29. “we searched”.ab. and (review.ti. or review.pt.)

30. “update review”.ab.

31. (databases adj4 searched).ab.

32. (rat or rats or mouse or mice or swine or porcine or murine or sheep or lambs or pigs or piglets or rabbit or rabbits or cat or cats or dog or dogs or cattle or bovine or monkey or monkeys or trout or marmoset$1).ti. and animal experiment/

33. Animal experiment/ not (human experiment/ or human/)

34. or/21-33

35. 20 not 34

36. electromagn$.ti,ab. or cortrak.ti,ab.

37. Endoscopy/exp or endoscop$.ti,ab.

38. “Enteral Nutrition”/exp or nasoenteral.ti,ab. or nasojejunal.ti,ab. or nasoduodenal.ti,ab. or postpyloric.ti,ab. or post-pyloric.ti,ab. or nasointestinal.ti,ab. or nasoenteric.ti,ab.

39. and/36-38

40. 35 and 39

CENTRAL

up to 30 September 2020.

1. electromagn*:ti,ab,kw

2. cortrak:ti,ab,kw

3. 1 or 2

4. [mh Endoscopy]

5. endoscop$:ti,ab,kw

6. 4 or 5

7. [mh “Enteral Nutrition”]

8. nasoenteral:ti,ab,kw

9. nasojejunal:ti,ab,kw

10. nasoduodenal:ti,ab,kw

11. postpyloric:ti,ab,kw

12. post-pyloric:ti,ab,kw

13. nasointestinal:ti,ab,kw

14. nasoenteric:ti,ab,kw

15. or/7-14
16. 3 and 6 and 15
