# Supplementary material for: Electromagnetic-guided versus endoscopic placement of nasoenteral feeding tubes: protocol for a systematic review and meta-analysis
Source: BMJ Open. 2021 Mar 10;11(3):e044637. doi: 10.1136/bmjopen-2020-044637 (PMC7949433; doi:10.1136/bmjopen-2020-044637)
Supplement: Author's manuscript [file bmjopen-2020-044637.draft_revisions.pdf]

BMJ Open

BMJ Open is committed to open peer review. As part of this commitment we make the peer review history of every article we publish publicly available.

When an article is published we post the peer reviewers' comments and the authors' responses online. We also post the versions of the paper that were used during peer review. These are the versions that the peer review comments apply to.

The versions of the paper that follow are the versions that were submitted during the peer review process. They are not the versions of record or the final published versions. They should not be cited or distributed as the published version of this manuscript.

BMJ Open is an open access journal and the full, final, typeset and author-corrected version of record of the manuscript is available on our site with no access controls, subscription charges or pay-per-view fees (http://bmjopen.bmj.com).

If you have any questions on BMJ Open's open peer review process please email info.bmjopen@bmj.com

BMJ Open

Electromagnetic-guided versus endoscopic placement of nasoenteral feeding tubes: protocol for a systematic review and meta-analysis

Journal:	*BMJ Open*
Manuscript ID	bmjopen-2020-044637
Article Type:	Protocol
Date Submitted by the Author:	09-Sep-2020
Complete List of Authors:	Jin, Zheng; Affiliated Hangzhou First People's Hospital Zhejiang University School of Medicine<br>Wei, Yaping; Affiliated Hangzhou First People's Hospital Zhejiang University School of Medicine<br>Shen, Guofan; Affiliated Hangzhou First People's Hospital Zhejiang University School of Medicine<br>Zhang, Xiaofeng; Affiliated Hangzhou First People's Hospital Zhejiang University School of Medicine
Keywords:	Endoscopy < GASTROENTEROLOGY, Nutritional support < GASTROENTEROLOGY, Adult intensive & critical care < INTENSIVE & CRITICAL CARE

SCHOLARONE™
Manuscripts

I, the Submitting Author has the right to grant and does grant on behalf of all authors of the Work (as defined in the below author licence), an exclusive licence and/or a non-exclusive licence for contributions from authors who are: i) UK Crown employees; ii) where BMJ has agreed a CC-BY licence shall apply, and/or iii) in accordance with the terms applicable for US Federal Government officers or employees acting as part of their official duties; on a worldwide, perpetual, irrevocable, royalty-free basis to BMJ Publishing Group Ltd ("BMJ") its licensees and where the relevant Journal is co-owned by BMJ to the co-owners of the Journal, to publish the Work in this journal and any other BMJ products and to exploit all rights, as set out in our licence.

The Submitting Author accepts and understands that any supply made under these terms is made by BMJ to the Submitting Author unless you are acting as an employee on behalf of your employer or a postgraduate student of an affiliated institution which is paying any applicable article publishing charge ("APC") for Open Access articles. Where the Submitting Author wishes to make the Work available on an Open Access basis (and intends to pay the relevant APC), the terms of reuse of such Open Access shall be governed by a Creative Commons licence – details of these licences and which Creative Commons licence will apply to this Work are set out in our licence referred to above.

Other than as permitted in any relevant BMJ Author's Self Archiving Policies, I confirm this Work has not been accepted for publication elsewhere, is not being considered for publication elsewhere and does not duplicate material already published. I confirm all authors consent to publication of this Work and authorise the granting of this licence.

For peer review only

Title Page

Title

Electromagnetic-guided versus endoscopic placement of nasoenteral feeding tubes: protocol for a systematic review and meta-analysis

Authors

Zheng Jin MD*, Yaping Wei MD*, Guofan Shen MD*, Xiaofeng Zhang MD

Affiliated Hangzhou First People's Hospital, Zhejiang University School of Medicine, Hangzhou, China

*These authors have contributed equally to this work.

Correspondence to: Xiaofeng Zhang, MD, Department of Gastroenterology, Affiliated Hangzhou First People's Hospital, Zhejiang University School of Medicine, #261 Huansha Road, Hangzhou, 310006, Zhejiang Province, China.

Fax: +86-0571-56006872

E-mail address: gangdaoersan@163.com

The word count excluding abstract was 1409.

For peer review only

ABSTRACT

Introduction: Current evidence supporting the utility of electromagnetic (EM)-guided method as the preferred technique for nasoenteral feeding tube placement is limited. We plan to provide a meta-analysis to compare the performance of EM-guided versus endoscopic placement.

Methods and analysis: Randomized controlled trials evaluating EM-guided vs. endoscopic placement will be searched in MEDLINE, EMBASE, Web of Science, the Cochrane Library, ClinicalTrials.gov and Google Scholar, from database inception to 30 September 2020. Data on study design, participant characteristics, intervention details and outcomes will be extracted. Primary outcomes to be assessed are procedure success rate. Secondary outcomes include reinsertion rate, number of attempts, placement-related complications, tube-related complications, insertion time, total procedure time, patient discomfort, patient recommendation, length of hospital stay, intensive care unit mortality, in-hospital mortality, and total costs. Study quality will be assessed using the Cochrane Risk of Bias Tool. Meta-analysis will be performed using RevMan V.5.3 statistical software. Data will be combined with a random effect model. The results will be

For peer review only

For peer review only - http://bmjopen.bmj.com/site/about/guidelines.xhtml

presented as a risk ratio for dichotomous data, and weighted/standard mean difference for continuous data. Publication bias will be visualized using funnel plots.

Ethics and dissemination: This study will not use primary data, and therefore formal ethical approval is not required. The findings will be disseminated through peer-reviewed journals and committee conferences.

PROSPERO registration number: CRD42020172427

ARTICLE SUMMARY

Strengths and limitations of this study

1. This is the first meta-analysis comparing electromagnetic-guided versus endoscopic placement of nasoenteral feeding tubes.

2. The study selection, data extraction and quality assessment will be performed independently by two researchers.

3. We will comprehensively survey the literature and identify areas where further study may be required.

4. A possible weakness may be the quality of the RCTs we include.

For peer review only

INTRODUCTION

Malnutrition and inability to eat are conditions often encountered in inpatients. For such patients, enteral nutrition is considered to be superior to parenteral nutrition since it reduces complications, improves patient outcome, and is more cheaper.[1, 2] It is common practice to place a nasoenteral feeding tube for enteral nutrition in patients who are intolerant of intragastric nutrition.[1, 2] Endoscopic technique is typically used, but requires patient transportation between wards, preprocedural fasting, and radiological confirmation of the tube' s position. Since first reported by Phang et al. in 2006,[4] electromagnetic (EM)-guided technique has been increasingly used for nasoenteral feeding tube placement. It has been suggested to be convenient and lead to a significant cost reduction. With increasing availability and familiarity with this technique, several randomized controlled trials (RCTs)[5-8] have compared EM-guided versus endoscopic (ENDO) technique. These studies have reported variable results and were limited because of small sample sizes. We had planned to conduct a meta-analysis to compare the performance between EM and ENDO.

METHODS

The review will be performed according to the recommendations specified in the Cochrane Handbook for Intervention Reviews.[9] The reporting of the review will follow

For peer review only - http://bmjopen.bmj.com/site/about/guidelines.xhtml

the Preferred Reporting Items for Systematic Reviews and Meta-Analysis (PRISMA) statement.[10]

Criteria for considering studies for this review

Eligibility criteria are established in terms of the Population-Intervention-Comparison-Outcome-Study design framework. Studies will be selected according to the following criteria:

Participants

Included studies will involve adult patients with an indication for enteral nutrition via a nasoenteral feeding tube, as indicated by the treating physician and/or consulting dietitian. Both critically ill and non-critically ill patients will be included. Exclusion criteria will be high suspicion of stenosis of the upper gastrointestinal tract, esophageal varices, signs of upper gastrointestinal hemorrhage and pregnancy.

Interventions/comparison

The intervention comparisons are EM vs. ENDO. Studies with additional prokinetic agent administration will also be included.

Outcomes

For peer review only - http://bmjopen.bmj.com/site/about/guidelines.xhtml

For peer review only

Primary outcome: procedure success rate (defined as the percentage of successful tube placement in the desired location as determined).

There are 12 secondary outcomes: reinsertion rate, number of attempts, placement-related complications, tube-related complications, insertion time, total procedure time, patient discomfort, patient recommendation, length of hospital stay, intensive care unit (ICU) mortality, in-hospital mortality, and total costs.

Study design

Only RCTs will be included. An abstract with sufficient data will also be considered. We will only include studies that are presented in English language due to constraints in translational resources.

Studies will be excluded if it meets at least one of the following criteria: (i) single arm study; (ii) case reports, reviews, editorials and letters to editor; (iii) duplicate studies, in vitro studies, or animal studies; (iv) no data on any of the primary or secondary outcomes.

Search methods for identification of studies

Electronic searches

Two investigators (ZJ and YW) will independently search MEDLINE, EMBASE, Web of Science, the Cochrane Library, for all entries through 30 September 2020 using the

For peer review only - http://bmjopen.bmj.com/site/about/guidelines.xhtml

following search terms: “Cortrak”, “electromagnetic”, “endoscopic”, “nasoenteral, or post-pyloric”, and “tube(s), feeding, or nutrition”. The search strategies will be decided on after a discussion among all reviewers. The primary search strategy will be used for PubMed MEDLINE (Online supplementary appendix I). Modifications to the search strategy will be made for other databases. We will assess eligibility of the retrieved articles by title and abstract using predetermined inclusion criteria. If this information is insufficient for eligibility assessment, we will review the full article. If any up-to-date evidence is published during the review period, we will evaluate the eligibility of each study and consider its addition to the analysis.

Searching other resources

To further increase the robustness of the literature search, a manual recursive search of the reference sections of the retrieved articles, as well as the related articles option in PubMed, will be carried out to identify other potentially relevant articles. Additional searches of ClinicalTrials.gov and Google Scholar will also be conducted.

Data collection and analysis

Selection of studies

Decisions about study inclusion and exclusion will be made independently by two investigators (ZJ and YW). Disagreements will be resolved by consensus after a mutual

For peer review only - http://bmjopen.bmj.com/site/about/guidelines.xhtml

discussion. The details of the study selection procedure are shown in a PRISMA flow chart. (Figure 1)

Data extraction and management

Two investigators (YW and GS) will independently extract the appropriate data onto Standardised extraction forms. The following data will be extracted from included trials: author, year of publication, country of origin, number of centers, participating operators, patient demographics, indications for enteral nutrition, and study outcomes. When necessary data are not included in the published papers, the first or corresponding authors will be contacted for additional information. If there is no reply, we will analyse only the available data. If there are no data on any of the primary or secondary outcomes, those studies will be excluded from the meta-analyses.

Assessment of risk of bias in included studies

We will assign two independent investigators (YW and GS) to appraise methodological quality of the included trials with the Cochrane Collaboration' s tool for assessing risk of bias.[11] The tool appraises existence of selection bias by assessing methods of randomization and allocation concealment, performance and detection of biases by checking blinding of personnel and outcome assessment, and attrition and reporting bias by evaluating incomplete and selective data reporting. Each of the item is assigned a judgment of high, low, or unclear risk.

For peer review only - http://bmjopen.bmj.com/site/about/guidelines.xhtml

Data synthesis

Risk ratios (RRs) will be calculated for categorical variables. Standard mean differences (SMDs) will be calculated for continuous variables including total costs based on different monetary units. Weighted mean differences (WMDs) will be calculated for continuous variables. Medians will be used if means are not available and standard deviations (SDs) will be calculated or imputed when possible.[12] Owning to the assumption of inherently various study scenarios and study populations, a random effects model for all analyses will be assumed. Heterogeneity among studies will be assessed by calculating the I^2 statistics whereby I^2<25% indicates no heterogeneity, 25%≤ I^2 <50% indicates mild heterogeneity, 50%≤ I^2 <75% indicates moderate heterogeneity and I^2 ≥75% indicates strong heterogeneity.[13] We had planned that if sufficient studies (≥10) are included in the analysis of primary outcomes, we would construct funnel plots to evaluate publication bias,[9] otherwise, Egger's test will be applied.[14] All statistical analyses will be performed using Review Manager 5.3 (The Cochrane Collaboration, The Nordic Cochrane Centre, Copenhagen, Denmark).

Subgroup analyses

In the case of possible strong heterogeneity, we will explore the possible sources using subgroup and meta-regression analyses. Subgroup analyses will be carried out based on geographical location, study setting, the BMI level of patients with the validated method

For peer review only - http://bmjopen.bmj.com/site/about/guidelines.xhtml

(ENDO group), prior altered upper gastrointestinal anatomy (with vs. without), patient population (critically ill vs. non-critically ill patients). For those subgroups with only 1 study included, subgroup analyses will not be performed.

Sensitivity analysis

We will carry out a sensitivity analysis by systematically removing every study and checking the pooled results for the remaining studies to see if there is any significant change in test performance.

Patient and public involvement

Because the collected data within this systematic review and meta-analysis originates from previously published studies, patients and the general public were not involved in the development of the research question or choice of outcome measures that we wanted to assess.

DISCUSSION

Conventional methods for the placement of nasoenteral feeding tubes include blind, fluoroscopic, and endoscopic methods.[15, 16] The gold standard for the placement of nasoenteral feeding tube is the endoscopic technique, which has success rates above 90%.[17, 18] Bed-side EM-guided tube placement can be performed in recent years. This has several potential advantages compared with endoscopic placement because only one

For peer review only - http://bmjopen.bmj.com/site/about/guidelines.xhtml

trained nurse and less equipment is needed. Several RCTs have compared EM-guided versus endoscopic technique with variable results.We therefore propose a meta-analysis to pool the evidence to further evaluate the performance of EM vs. ENDO.

This will be the first meta-analysis comparing EM with ENDO for the placement of nasoenteral feeding tube. The results of this study will influence decision making for the patients who are malnutrition or inability to eat, assist in future guideline development and guide future research endeavors.

ETHICS AND DISSEMINATION

Ethics approval and patient written informed consent will not be required because all analyses in the present study will be performed based on data from published studies. We will disseminate the findings of our work through conference presentations and a peer-reviewed publication.

AUTHOR STATEMENT

Author Contributions

ZJ and XZ conceived and designed this study. ZJ and YW searched and selected the studies. YW and GS extracted the essential information. YW and GS assessed the risk of bias. GS and ZJ performed the statistical analyses. ZJ and YW interpreted the pooled

For peer review only - http://bmjopen.bmj.com/site/about/guidelines.xhtml

results. ZJ, YW and GS drafted the manuscript. All authors approved the manuscript to be considered for publication.

Competing interests: None.

Funding: None.

For peer review only

For peer review only

References:

1. Doig GS, Heighes PT, Simpson F, Sweetman EA, Davies AR. Early enteral nutrition, provided within 24 h of injury or intensive care unit admission, significantly reduces mortality in critically ill patients: a meta-analysis of randomised controlled trials. *Intensive Care Med*. 2009; 35: 2018-27.

2. Jones NE, Heyland DK. Implementing nutrition guidelines in the critical care setting: a worthwhile and achievable goal? *JAMA*. 2008; 300: 2798-9.

3. Hsu CW, Sun SF, Lin SL, et al. Duodenal versus gastric feeding in medical intensive care unit patients: a prospective, randomized, clinical study. *Crit. Care Med.* 2009; 37: 1866-72.

4. Phang J, Marsh W, Prager R. Feeding Tube Placement with the Aid of a New Electromagnetic Transmitter.: S082. *Jpen.-Parenter. Enter.* 2006; 30.

For peer review only - http://bmjopen.bmj.com/site/about/guidelines.xhtml

5. Gao X, Zhang L, Zhao J, et al. Bedside electromagnetic-guided placement of nasoenteral feeding tubes among critically Ill patients: A single-centre randomized controlled trial. *J. Crit. Care*. 2018; 48: 216-21.

6. Gerritsen A, de Rooij T, Dijkgraaf MG, et al. Electromagnetic-Guided Bedside Placement of Nasoenteral Feeding Tubes by Nurses Is Non-Inferior to Endoscopic Placement by Gastroenterologists: A Multicenter Randomized Controlled Trial. *Am. J. Gastroenterol.* 2016; 111: 1123-32.

7. Holzinger U, Brunner R, Miehsler W, et al. Jejunal tube placement in critically ill patients: A prospective, randomized trial comparing the endoscopic technique with the electromagnetically visualized method. *Crit. Care Med.* 2011; 39: 73-7.

8. Kappelle W, Walter D, Stadhouders PH, et al. Electromagnetic-guided placement of nasoduodenal feeding tubes versus endoscopic placement: a randomized, multicenter trial. *Gastrointest. Endosc.* 2018; 87: 110-8.

9. Higgins JP, Green S. Cochrane Handbook for Systematic Reviews of Interventions.; 2011.

10. Moher D, Liberati A, Tetzlaff J, Altman DG. Preferred reporting items for systematic reviews and meta-analyses: the PRISMA statement. *BMJ*. 2009; 339: b2535.

11. Higgins JP, Altman DG, Gotzsche PC, et al. The Cochrane Collaboration's tool for assessing risk of bias in randomised trials. *BMJ*. 2011; 343: d5928.

12. Hozo SP, Djulbegovic B, Hozo I. Estimating the mean and variance from the median, range, and the size of a sample. *BMC Med. Res. Methodol.* 2005; 5: 13.

13. Higgins JP, Thompson SG. Quantifying heterogeneity in a meta-analysis. *Stat. Med.* 2002; 21: 1539-58.

14. Egger M, Davey SG, Schneider M, Minder C. Bias in meta-analysis detected by a simple, graphical test. *BMJ*. 1997; 315: 629-34.

15. Hillard AE, Waddell JJ, Metzler MH, McAlpin D. Fluoroscopically guided nasoenteric feeding tube placement versus bedside placement. *South. Med. J.* 1995; 88: 425-8.

16. Scott R, Bowling TE. Enteral tube feeding in adults. *J R Coll Physicians Edinb*. 2015; 45: 49-54.

17. Byrne KR, Fang JC. Endoscopic placement of enteral feeding catheters. *Curr Opin Gastroenterol*. 2006; 22: 546-50.

For peer review only - http://bmjopen.bmj.com/site/about/guidelines.xhtml

18. Wiggins TF, DeLegge MH. Evaluation of a new technique for endoscopic nasojejunal feeding-tube placement. *Gastrointest. Endosc.* 2006; 63: 590-5.

19. Kline AM, Sorce L, Sullivan C, Weishaar J, Steinhorn DM. Use of a Noninvasive Electromagnetic Device to Place Transpyloric Feeding Tubes in Critically Ill Children. *Am. J. Crit. Care*. 2011; 20: 453-60.

20. Arjaans W, Ouwehand M, Bouma G, van der Meulen T, de van der Schueren M. Cortrak((R)) duodenal tube placements: A solution for more patients? A preliminary survey to the introduction of electromagnetic-guided placement of naso-duodenal feeding tubes. *Clin Nutr ESPEN*. 2019; 29: 133-6.

For peer review only

Figure 1: Flow diagram of the study selection process.

For peer review only - http://bmjopen.bmj.com/site/about/guidelines.xhtml

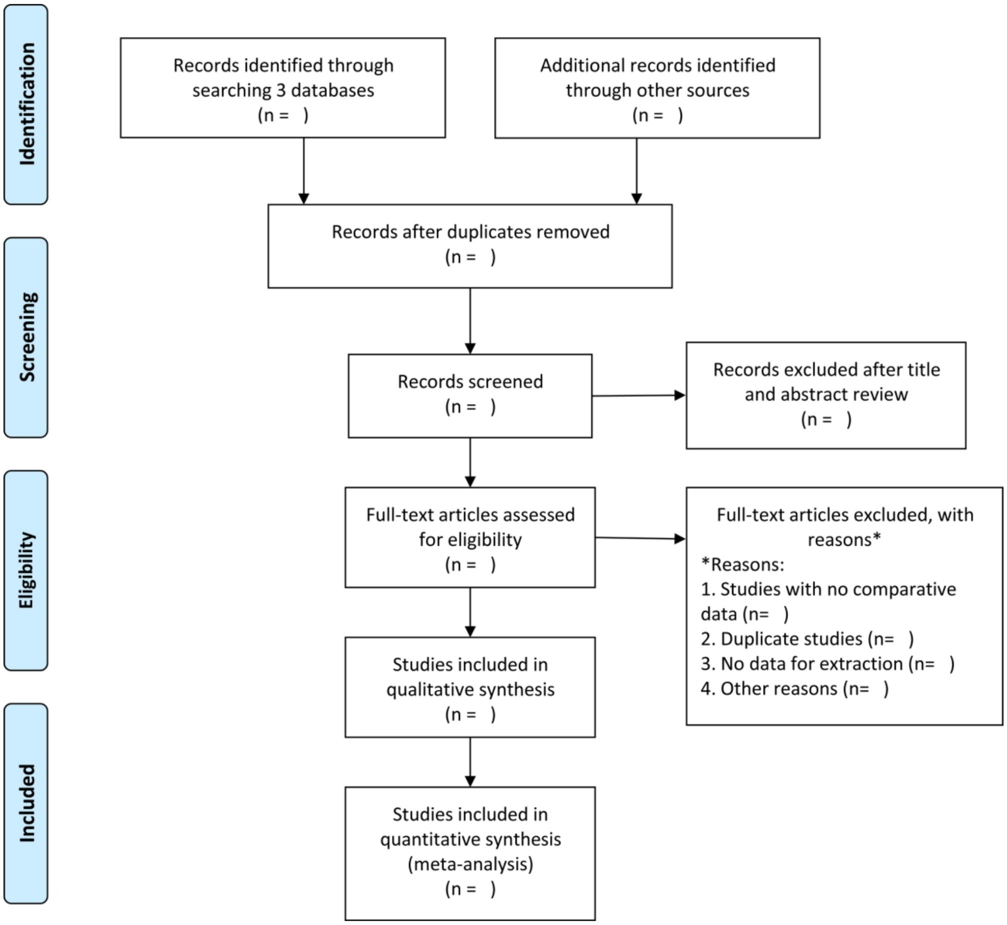

Flow diagram of the study selection process.

190x174mm (300 x 300 DPI)

Online supplementary appendix 1. PubMed-MEDLINE search strategy

up to 30 September 2020.

#1 electromagn*[tiab] OR cortrak[tiab]

#2 endoscop*[tiab]

#3 #1 AND #2

#4 nasoenteral[tiab] OR nasojejunal[tiab] OR nasoduodenal[tiab] OR postpyloric[tiab] OR post-pyloric[tiab] OR nasointestinal[tiab] OR nasoenteric[tiab]

#5 "randomized controlled trial"[pt] OR "controlled clinical trial"[pt] OR "randomized"[tiab] OR "randomised"[tiab] OR "randomly"[tiab]

#6 #3 AND #4 AND #5

For peer review only

1

PRISMA-P 2015 Checklist

This checklist has been adapted for use with protocol submissions to *Systematic Reviews* from Table 3 in Moher D et al: Preferred reporting items for systematic review and meta-analysis protocols (PRISMA-P) 2015 statement. *Systematic Reviews* 2015 **4**:1

Section/topic	#	Checklist item	Information reported Yes	Information reported No	Line number(s)
**ADMINISTRATIVE INFORMATION**					
**Title**					
Identification	1a	Identify the report as a protocol of a systematic review	☒	☐	Title
Update	1b	If the protocol is for an update of a previous systematic review, identify as such	☐	☒	Not applicable
**Registration**	2	If registered, provide the name of the registry (e.g., PROSPERO) and registration number in the Abstract	☒	☐	Abstract
**Authors**					
Contact	3a	Provide name, institutional affiliation, and e-mail address of all protocol authors; provide physical mailing address of corresponding author	☒	☐	Page 1
Contributions	3b	Describe contributions of protocol authors and identify the guarantor of the review	☒	☐	Page 11
**Amendments**	4	If the protocol represents an amendment of a previously completed or published protocol, identify as such and list changes; otherwise, state plan for documenting important protocol amendments	☐	☒	Not applicable
**Support**					
Sources	5a	Indicate sources of financial or other support for the review	☐	☒	Not applicable
Sponsor	5b	Provide name for the review funder and/or sponsor	☐	☒	Not applicable
Role of sponsor/funder	5c	Describe roles of funder(s), sponsor(s), and/or institution(s), if any, in developing the protocol	☐	☒	Not applicable
**INTRODUCTION**					
**Rationale**	6	Describe the rationale for the review in the context of what is already known	☒	☐	Page 4
**Objectives**	7	Provide an explicit statement of the question(s) the review will address with reference to participants, interventions, comparators, and outcomes (PICO)	☒	☐	Page 4
**METHODS**					
**Eligibility criteria**	8	Specify the study characteristics (e.g., PICO, study design, setting, time frame) and report characteristics (e.g., years considered, language, publication status) to be used as criteria for eligibility for the review	☒	☐	Page 4

BioMed Central The Open Access Publisher

Section/topic	#	Checklist item	Information reported: Yes	Information reported: No	Line number(s)
**Information sources**	9	Describe all intended information sources (e.g., electronic databases, contact with study authors, trial registers, or other grey literature sources) with planned dates of coverage	☒	☐	Page 6
**Search strategy**	10	Present draft of search strategy to be used for at least one electronic database, including planned limits, such that it could be repeated	☒	☐	Online supplementary appendix I
STUDY RECORDS					
Data management	11a	Describe the mechanism(s) that will be used to manage records and data throughout the review	☒	☐	Page 7-8
Selection process	11b	State the process that will be used for selecting studies (e.g., two independent reviewers) through each phase of the review (i.e., screening, eligibility, and inclusion in meta-analysis)	☒	☐	Page 7-8
Data collection process	11c	Describe planned method of extracting data from reports (e.g., piloting forms, done independently, in duplicate), any processes for obtaining and confirming data from investigators	☒	☐	Page 7-8
**Data items**	12	List and define all variables for which data will be sought (e.g., PICO items, funding sources), any pre-planned data assumptions and simplifications	☒	☐	Page 6
**Outcomes and prioritization**	13	List and define all outcomes for which data will be sought, including prioritization of main and additional outcomes, with rationale	☒	☐	Page 6
**Risk of bias in individual studies**	14	Describe anticipated methods for assessing risk of bias of individual studies, including whether this will be done at the outcome or study level, or both; state how this information will be used in data synthesis	☒	☐	Page 8
DATA					
**Synthesis**	15a	Describe criteria under which study data will be quantitatively synthesized	☒	☐	Page 9
	15b	If data are appropriate for quantitative synthesis, describe planned summary measures, methods of handling data, and methods of combining data from studies, including any planned exploration of consistency (e.g., I^2, Kendall's tau)	☒	☐	Page 9
	15c	Describe any proposed additional analyses (e.g., sensitivity or subgroup analyses, meta-regression)	☒	☐	Page 9-10
	15d	If quantitative synthesis is not appropriate, describe the type of summary planned	☒	☐	
**Meta-bias(es)**	16	Specify any planned assessment of meta-bias(es) (e.g., publication bias across studies, selective reporting within studies)	☒	☐	Page 10
**Confidence in cumulative evidence**	17	Describe how the strength of the body of evidence will be assessed (e.g., GRADE)	☐	☒	

For peer review only

BioMed Central The Open Access Publisher

BMJ Open

Electromagnetic-guided versus endoscopic placement of nasoenteral feeding tubes: protocol for a systematic review and meta-analysis

Journal:	*BMJ Open*
Manuscript ID	bmjopen-2020-044637.R1
Article Type:	Protocol
Date Submitted by the Author:	14-Dec-2020
Complete List of Authors:	Jin, Zheng; Affiliated Hangzhou First People's Hospital Zhejiang University School of Medicine<br>Wei, Yaping; Affiliated Hangzhou First People's Hospital Zhejiang University School of Medicine<br>Shen, Guofan; Affiliated Hangzhou First People's Hospital Zhejiang University School of Medicine<br>Zhang, Xiaofeng; Affiliated Hangzhou First People's Hospital Zhejiang University School of Medicine
**Primary Subject Heading**:	Emergency medicine
Secondary Subject Heading:	Gastroenterology and hepatology
Keywords:	Endoscopy < GASTROENTEROLOGY, Nutritional support < GASTROENTEROLOGY, Adult intensive & critical care < INTENSIVE & CRITICAL CARE

SCHOLARONE™
Manuscripts

I, the Submitting Author has the right to grant and does grant on behalf of all authors of the Work (as defined in the below author licence), an exclusive licence and/or a non-exclusive licence for contributions from authors who are: i) UK Crown employees; ii) where BMJ has agreed a CC-BY licence shall apply, and/or iii) in accordance with the terms applicable for US Federal Government officers or employees acting as part of their official duties; on a worldwide, perpetual, irrevocable, royalty-free basis to BMJ Publishing Group Ltd ("BMJ") its licensees and where the relevant Journal is co-owned by BMJ to the co-owners of the Journal, to publish the Work in this journal and any other BMJ products and to exploit all rights, as set out in our licence.

The Submitting Author accepts and understands that any supply made under these terms is made by BMJ to the Submitting Author unless you are acting as an employee on behalf of your employer or a postgraduate student of an affiliated institution which is paying any applicable article publishing charge ("APC") for Open Access articles. Where the Submitting Author wishes to make the Work available on an Open Access basis (and intends to pay the relevant APC), the terms of reuse of such Open Access shall be governed by a Creative Commons licence – details of these licences and which Creative Commons licence will apply to this Work are set out in our licence referred to above.

Other than as permitted in any relevant BMJ Author's Self Archiving Policies, I confirm this Work has not been accepted for publication elsewhere, is not being considered for publication elsewhere and does not duplicate material already published. I confirm all authors consent to publication of this Work and authorise the granting of this licence.

For peer review only

Title Page

Title

Electromagnetic-guided versus endoscopic placement of nasoenteral feeding tubes: protocol for a systematic review and meta-analysis

Authors

Zheng Jin MD*, Yaping Wei MD*, Guofan Shen MD*, Xiaofeng Zhang MD

Affiliated Hangzhou First People's Hospital, Zhejiang University School of Medicine, Hangzhou, China

*These authors have contributed equally to this work.

Correspondence to: Xiaofeng Zhang, MD, Department of Gastroenterology, Affiliated Hangzhou First People's Hospital, Zhejiang University School of Medicine, #261 Huansha Road, Hangzhou, 310006, Zhejiang Province, China.

Fax: +86-0571-56006872

E-mail address: gangdaoersan@163.com

For peer review only

ABSTRACT

Introduction: Current evidence supporting the utility of electromagnetic (EM)-guided method as the preferred technique for nasoenteral feeding tube placement is limited. We plan to provide a meta-analysis to compare the performance of EM-guided versus endoscopic placement.

Methods and analysis: Randomized controlled trials evaluating EM-guided vs. endoscopic placement will be searched in MEDLINE, EMBASE, and CENTRAL, from database inception to 30 September 2020. Data on study design, participant characteristics, intervention details and outcomes will be extracted. Primary outcomes to be assessed are complications. Secondary outcomes include procedure success rate, total procedure time, patient recommendation, length of hospital stay, mortality, and total costs. Study quality will be assessed using the Cochrane Risk of Bias Tool. Data will be combined with a random effect model. The results will be presented as a risk ratio for dichotomous data, and weighted/standard mean difference for continuous data. Publication bias will be visualized using funnel plots. We will quantify the effect of potential effect modifiers by meta-regression if appropriate. The quality of evidence will be evaluated according to the GRADE framework.

For peer review only - http://bmjopen.bmj.com/site/about/guidelines.xhtml

Ethics and dissemination: This study will not use primary data, and therefore formal ethical approval is not required. The findings will be disseminated through peer-reviewed journals and committee conferences.

PROSPERO registration number: CRD42020172427

For peer review only

ARTICLE SUMMARY

Strengths and limitations of this study

1. This systematic review and meta-analysis will update the existing clinical evidence of placement of nasoenteral feeding tubes.

2. The thorough and transparent methodological approach undertaken will minimise the risk of possible biases. Quality of evidence will be assessed to provide confidence in the effect estimates.

3. Multiple methods for investigating heterogeneity can inform intervention and study design, but contingent on the number and size of available studies.

4. Common to any meta-analysis, some heterogeneity across and within studies may exist.

For peer review only - http://bmjopen.bmj.com/site/about/guidelines.xhtml

INTRODUCTION

Malnutrition and inability to eat are conditions often encountered in inpatients. For such patients, enteral nutrition is considered to be superior to parenteral nutrition since it reduces complications, improves patient outcome, and is more cheaper.[1, 2]

It is common practice to place a nasoenteral feeding tube for enteral nutrition in patients who are intolerant of intragastric nutrition.[3] Endoscopic technique is typically used, but requires patient transportation between wards, preprocedural fasting, and radiological confirmation of the tube' s position. Since first reported by Phang et al. in 2006,[4] electromagnetic (EM)-guided technique has been increasingly used for nasoenteral feeding tube placement. It has been suggested to be convenient and lead to a significant cost reduction. With increasing availability and familiarity with this technique, several randomized controlled trials (RCTs)[5-8] have compared EM-guided versus endoscopic (ENDO) technique. EM and ENDO technique have been compared in only one systematic review until now.[9] That review by Gerritsen et al. involving only one relevant RCT (66 patients) concluded that the efficacy and safety of the two techniques did not differ significantly, but EM offered advantages in logistics. In view of several new RCTs published, we have planned to pool the evidence to further evaluate the performance of EM vs. ENDO.

METHODS

For peer review only - http://bmjopen.bmj.com/site/about/guidelines.xhtml

The review will be performed according to the recommendations specified in the Cochrane Handbook for Intervention Reviews.[10] The reporting of the review will follow the Preferred Reporting Items for Systematic Reviews and Meta-Analysis (PRISMA) statement.[11]

Criteria for considering studies for this review

Eligibility criteria are established in terms of the Population-Intervention-Comparison-Outcome-Study design framework. Studies will be selected according to the following criteria:

Participants

Included studies will involve adult patients with an indication for enteral nutrition via a nasoenteral feeding tube, as indicated by the treating physician and/or consulting dietitian. Both critically ill and non-critically ill patients will be included. Exclusion criteria will be high suspicion of stenosis of the upper gastrointestinal tract, esophageal varices, signs of upper gastrointestinal hemorrhage and pregnancy.

Interventions/comparison

The intervention comparisons are EM vs. ENDO. Studies with additional prokinetic agent administration will also be included.

Outcomes

For peer review only - http://bmjopen.bmj.com/site/about/guidelines.xhtml

Primary outcome: complications.

There are 6 secondary outcomes: procedure success rate (defined as the percentage of successful tube placement in the desired location as determined), total procedure time, patient recommendation, length of hospital stay, mortality, and total costs (including the costs for tube placement procedure, complications and therapeutic interventions).

Study design

Only RCTs (including factorial, cross-over, sequential design, etc.) will be included. An abstract with sufficient data will also be considered. We will only include studies that are presented in English language due to constraints in translational resources.

Studies will be excluded if it meets at least one of the following criteria: (i) observational studies including case-control and cohort studies; (ii) single arm studies; (iii) case reports, reviews, editorials and letters to editor; (iv) duplicate studies, in vitro studies, or animal studies; (v) no data on any of the primary or secondary outcomes.

Search methods for identification of studies

Electronic searches

Two investigators (ZJ and YW) will independently search MEDLINE, EMBASE, and CENTRAL, for all entries through 30 September 2020 using the following search terms: “Cortrak”, “electromagnetic”, “endoscopic”, “nasoenteral, or post-pyloric”, and “tube(s),

For peer review only - http://bmjopen.bmj.com/site/about/guidelines.xhtml

feeding, or nutrition". The search strategies (Online supplementary appendix I) will be decided on after a discussion among all reviewers. We will assess eligibility of the retrieved articles by title and abstract using predetermined inclusion criteria. If this information is insufficient for eligibility assessment, we will review the full article. If any up-to-date evidence is published during the review period, we will evaluate the eligibility of each study and consider its addition to the analysis. To further increase the robustness of the literature search, a manual recursive search of the Web of Science for a list of studies that cite the included studies and studies that the included studies cited, will be carried out to identify other potentially relevant articles.

Data collection and analysis

Selection of studies

Decisions about study inclusion and exclusion will be made independently by two investigators (ZJ and YW). Disagreements will be resolved by consensus after a mutual discussion. The details of the study selection procedure are shown in a PRISMA flow chart. (Figure 1)

Data extraction and management

Two investigators (YW and GS) will independently extract the appropriate data onto Standardised extraction forms. The following data will be extracted from included trials: author, year of publication, country of origin, number of centers, participating operators,

For peer review only - http://bmjopen.bmj.com/site/about/guidelines.xhtml

patient demographics, indications for enteral nutrition, and study outcomes. When necessary data are not included in the published papers, the first or corresponding authors will be contacted for additional information. If there is no reply, we will analyse only the available data. If there are no data on any of the primary or secondary outcomes, those studies will be excluded from the meta-analyses.

For peer review only

Assessment of risk of bias in included studies

We will assign two independent investigators (YW and GS) to appraise methodological quality of the included trials with the Cochrane Collaboration' s tool for assessing risk of bias.[12] The tool appraises existence of selection bias by assessing methods of randomization and allocation concealment, performance and detection of biases by checking blinding of personnel and outcome assessment, and attrition and reporting bias by evaluating incomplete and selective data reporting. Each of the item is assigned a judgment of high, low, or unclear risk.

Data synthesis

Risk ratios (RRs) will be calculated for categorical variables. Standard mean differences (SMDs) will be calculated for continuous variables including total costs based on different monetary units. Weighted mean differences (WMDs) will be calculated for continuous variables. Medians will be used if means are not available and standard deviations (SDs) will be calculated or imputed when possible.[13] Owning to the

assumption of inherently various study scenarios and study populations, a random effects model for all analyses will be assumed. Heterogeneity among studies will be assessed by calculating the I^2 statistics whereby I^2<25% indicates no heterogeneity, 25%≤ I^2 <50% indicates mild heterogeneity, 50%≤ I^2 <75% indicates moderate heterogeneity and I^2 ≥75% indicates strong heterogeneity.[14] We had planned that if sufficient studies (≥10) are included in the analysis of primary outcomes, we would construct funnel plots to evaluate publication bias.[15] Egger test for low sample size with a significance level of 0.05, and Begg and Mazumdar test for high sample volume at a significance level of 0.1.[16] All statistical analyses will be performed using Review Manager 5.4.1 (The Cochrane Collaboration, The Nordic Cochrane Centre, Copenhagen, Denmark).

Meta-regression analysis

A random-effects meta-regression analysis will be conducted to elucidate the impact of core set covariates (mean age, the proportion of males, sample size,research year and risk of bias) on treatment effects.

Subgroup analyses

In the case of possible strong heterogeneity, we will explore the possible sources using subgroup analyses. It is reported that a higher body mass index (BMI) were associated with a successful placement for EM.[5, 7] So, subgroup analyses will be carried out based on geographical location, study setting, the BMI level of patients with the validated

For peer review only - http://bmjopen.bmj.com/site/about/guidelines.xhtml

method (ENDO group), prior altered upper gastrointestinal anatomy (with vs. without), patient population (critically ill vs. non-critically ill patients). For those subgroups with only 1 study included, subgroup analyses will not be performed.

Sensitivity analysis

We will carry out a sensitivity analysis by systematically removing every study and checking the pooled results for the remaining studies to see if there is any significant change in test performance.

Summary of evidence

We will summarise the quality of evidence using the GRADE (Grading of Recommendations Assessment, Development and Evaluation) approach and present 'Summary of findings' tables.

Patient and public involvement

Because the collected data within this systematic review and meta-analysis originates from previously published studies, patients and the general public were not involved in the development of the research question or choice of outcome measures that we wanted to assess.

DISCUSSION

For peer review only - http://bmjopen.bmj.com/site/about/guidelines.xhtml

Conventional methods for the placement of nasoenteral feeding tubes include blind, fluoroscopic, and endoscopic methods.[17, 18] The gold standard for the placement of nasoenteral feeding tube is the endoscopic technique, which has success rates above 90%.[19, 20] Bed-side EM-guided tube placement can be performed in recent years. This has several potential advantages compared with endoscopic placement because only one trained nurse and less equipment is needed.[21, 22] But current evidence supporting the utility of EM-guided method as the preferred technique is limited. We therefore propose an updated meta-analysis to pool the evidence to further evaluate the performance of EM vs. ENDO. The results of this study will influence decision making for the patients who are malnutrition or inability to eat, assist in future guideline development and guide future research endeavors.

ETHICS AND DISSEMINATION

Ethics approval and patient written informed consent will not be required because all analyses in the present study will be performed based on data from published studies. We will disseminate the findings of our work through conference presentations and a peer-reviewed publication.

AUTHOR STATEMENT

Author Contributions

For peer review only - http://bmjopen.bmj.com/site/about/guidelines.xhtml

ZJ and XZ conceived and designed this study. ZJ and YW searched and selected the studies. YW and GS extracted the essential information. YW and GS assessed the risk of bias. GS and ZJ performed the statistical analyses. ZJ and YW interpreted the pooled results. ZJ, YW and GS drafted the manuscript. All authors approved the manuscript to be considered for publication.

Competing interests: None.

Funding: None.

For peer review only

References:

1. Doig GS, Heighes PT, Simpson F, Sweetman EA, Davies AR. Early enteral nutrition, provided within 24 h of injury or intensive care unit admission, significantly reduces mortality in critically ill patients: a meta-analysis of randomised controlled trials. *Intensive Care Med*. 2009; **35**: 2018-27.

2. Jones NE, Heyland DK. Implementing nutrition guidelines in the critical care setting: a worthwhile and achievable goal? *JAMA*. 2008; **300**: 2798-9.

3. Hsu CW, Sun SF, Lin SL*, et al.*. Duodenal versus gastric feeding in medical intensive care unit patients: a prospective, randomized, clinical study. *CRIT CARE MED*. 2009; **37**: 1866-72.

4. Phang J, Marsh W, Prager R. Feeding Tube Placement with the Aid of a New Electromagnetic Transmitter. *JPEN-PARENTER ENTER*. 2006; **30**: S82.

5. Gao X, Zhang L, Zhao J*, et al.*. Bedside electromagnetic-guided placement of nasoenteral feeding tubes among critically Ill patients: A single-centre randomized controlled trial. *J CRIT CARE*. 2018; **48**: 216-21.

For peer review only - http://bmjopen.bmj.com/site/about/guidelines.xhtml

6. Gerritsen A, de Rooij T, Dijkgraaf MG, *et al.*. Electromagnetic-Guided Bedside Placement of Nasoenteral Feeding Tubes by Nurses Is Non-Inferior to Endoscopic Placement by Gastroenterologists: A Multicenter Randomized Controlled Trial. *AM J GASTROENTEROL*. 2016; **111**: 1123-32.

7. Holzinger U, Brunner R, Miehsler W, *et al.*. Jejunal tube placement in critically ill patients: A prospective, randomized trial comparing the endoscopic technique with the electromagnetically visualized method. *CRIT CARE MED*. 2011; **39**: 73-7.

8. Kappelle W, Walter D, Stadhouders PH, *et al.*. Electromagnetic-guided placement of nasoduodenal feeding tubes versus endoscopic placement: a randomized, multicenter trial. *GASTROINTEST ENDOSC*. 2018; **87**: 110-8.

9. Gerritsen A, van der Poel MJ, de Rooij T, *et al.*. Systematic review on bedside electromagnetic-guided, endoscopic, and fluoroscopic placement of nasoenteral feeding tubes. *GASTROINTEST ENDOSC*. 2015; **81**: 836-47.

10. Higgins JPT, Thomas J, Chandler J, *et al.*. Cochrane Handbook for Systematic Reviews of Interventions version 6.1., 2020.

11. Moher D, Liberati A, Tetzlaff J, Altman DG. Preferred reporting items for systematic reviews and meta-analyses: the PRISMA statement. *BMJ*. 2009; **339**: b2535.

12. Higgins JP, Altman DG, Gotzsche PC, *et al.*. The Cochrane Collaboration's tool for assessing risk of bias in randomised trials. *BMJ*. 2011; **343**: d5928.

13. Hozo SP, Djulbegovic B, Hozo I. Estimating the mean and variance from the median, range, and the size of a sample. *BMC MED RES METHODOL*. 2005; **5**: 13.

14. Higgins JP, Thompson SG. Quantifying heterogeneity in a meta-analysis. *STAT MED*. 2002; **21**: 1539-58.

15. Higgins JP, Green S. Cochrane Handbook for Systematic Reviews of Interventions., 2011.

16. Egger M, Davey SG, Schneider M, Minder C. Bias in meta-analysis detected by a simple, graphical test. *BMJ*. 1997; **315**: 629-34.

17. Hillard AE, Waddell JJ, Metzler MH, McAlpin D. Fluoroscopically guided nasoenteric feeding tube placement versus bedside placement. *SOUTH MED J*. 1995; **88**: 425-8.

18. Scott R, Bowling TE. Enteral tube feeding in adults. *J R Coll Physicians Edinb*. 2015; **45**: 49-54.

For peer review only - http://bmjopen.bmj.com/site/about/guidelines.xhtml

19. Byrne KR, Fang JC. Endoscopic placement of enteral feeding catheters. *Curr Opin Gastroenterol*. 2006; **22**: 546-50.

20. Wiggins TF, DeLegge MH. Evaluation of a new technique for endoscopic nasojejunal feeding-tube placement. *GASTROINTEST ENDOSC*. 2006; **63**: 590-5.

21. Kline AM, Sorce L, Sullivan C, Weishaar J, Steinhorn DM. Use of a Noninvasive Electromagnetic Device to Place Transpyloric Feeding Tubes in Critically Ill Children. *AM J CRIT CARE*. 2011; **20**: 453-60.

22. Arjaans W, Ouwehand M, Bouma G, van der Meulen T, de van der Schueren M. Cortrak((R)) duodenal tube placements: A solution for more patients? A preliminary survey to the introduction of electromagnetic-guided placement of naso-duodenal feeding tubes. *Clin Nutr ESPEN*. 2019; **29**: 133-6.

For peer review only

Figure 1. Flow diagram of the study selection process.

Online supplementary appendix 1: Search strategies

For peer review only

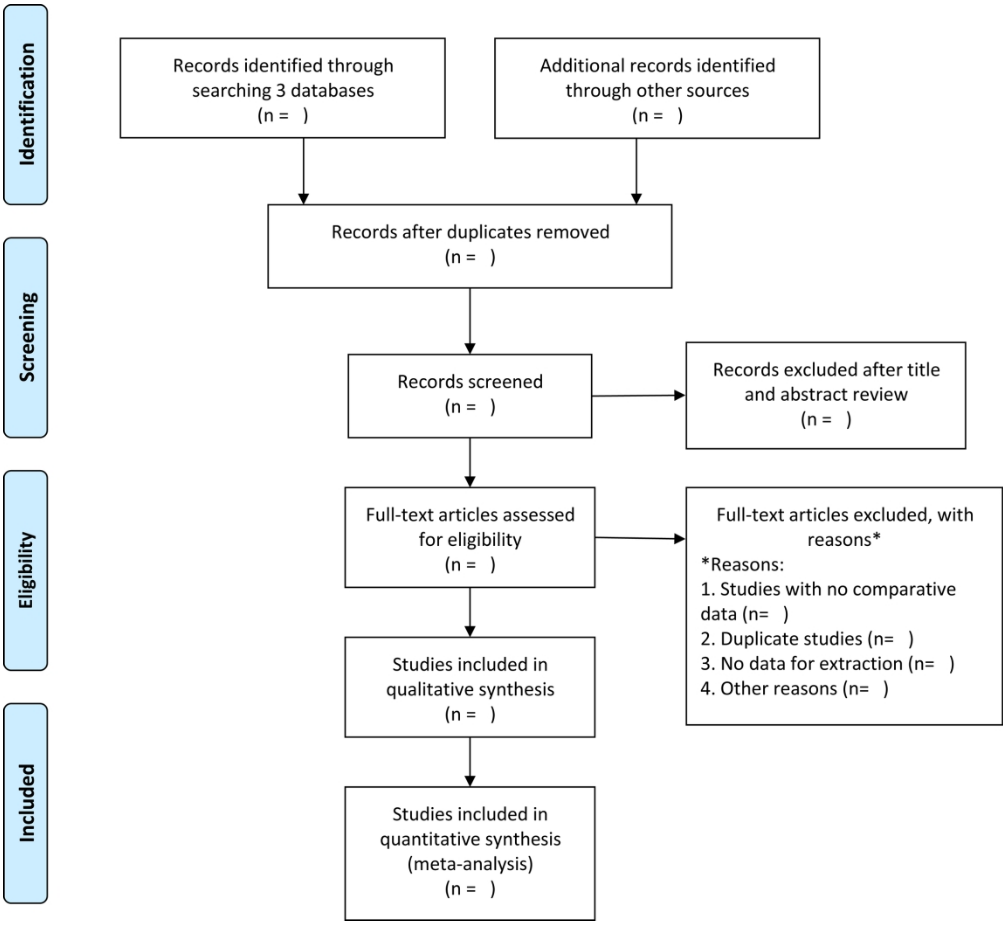

Flow diagram of the study selection process.

190x174mm (300 x 300 DPI)

Online supplementary appendix 1. Search strategies

MEDLINE (PubMed format)

up to 30 September 2020.

1. electromagn* [tiab] OR cortrak [tiab]
2. Endoscopy [mesh] OR endoscop* [tiab]
3. Enteral Nutrition [mesh] OR nasoenteral [tiab] OR nasojejunal [tiab] OR nasoduodenal [tiab] OR postpyloric [tiab] OR post-pyloric [tiab] OR nasointestinal [tiab] OR nasoenteric [tiab]
4. randomized controlled trial [pt] OR controlled clinical trial [pt] OR randomized [tiab] OR placebo [tiab] OR clinical trials as topic [mesh: noexp] OR randomly [tiab] OR trial [ti]
5. animals [mesh] NOT humans [mesh]
6. 4 NOT 5
7. 1 AND 2 AND 3 AND 6

EMBASE (Ovid format)

up to 30 September 2020.

1. Randomized controlled trial/
2. Controlled clinical study/
3. random$.ti,ab.
4. randomization/
5. intermethod comparison/

For peer review only

6. placebo.ti,ab.

7. (compare or compared or comparison).ti.

8. ((evaluated or evaluate or evaluating or assessed or assess) and (compare or compared or comparing or comparison)).ab.

9. (open adj label).ti,ab.

10. ((double or single or doubly or singly) adj (blind or blinded or blindly)).ti,ab.

11. double blind procedure/

12. parallel group$1.ti,ab.

13. (crossover or cross over).ti,ab.

14. ((assign$ or match or matched or allocation) adj5 (alternate or group$1 or intervention$1 or patient$1 or subject$1 or participant$1)).ti,ab.

15. (assigned or allocated).ti,ab.

16. (controlled adj7 (study or design or trial)).ti,ab.

17. (volunteer or volunteers).ti,ab.

18. human experiment/

19. trial.ti.

20. or/1-19

21. random$ adj sampl$ adj7 ("cross section$" or questionnaire$1 or survey$ or database$1)).ti,ab. not (comparative study/ or controlled study/ or randomi?ed controlled.ti,ab. or randomly assigned.ti,ab.)

22. Cross-sectional study/ not (randomized controlled trial/ or controlled clinical study/ or controlled study/ or randomi?ed controlled.ti,ab. or control group$1.ti,ab.)

For peer review only

23. (((case adj control$) and random$) not randomi?ed controlled).ti,ab.

24. (Systematic review not (trial or study)).ti.

25. (nonrandom$ not random$).ti,ab.

26. "Random field$".ti,ab.

27. (random cluster adj3 sampl$).ti,ab.

28. (review.ab. and review.pt.) not trial.ti.

29. "we searched".ab. and (review.ti. or review.pt.)

30. "update review".ab.

31. (databases adj4 searched).ab.

32. (rat or rats or mouse or mice or swine or porcine or murine or sheep or lambs or pigs or piglets or rabbit or rabbits or cat or cats or dog or dogs or cattle or bovine or monkey or monkeys or trout or marmoset$1).ti. and animal experiment/

33. Animal experiment/ not (human experiment/ or human/)

34. or/21-33

35. 20 not 34

36. electromagn$.ti,ab. or cortrak.ti,ab.

37. Endoscopy/exp or endoscop$.ti,ab.

38. "Enteral Nutrition"/exp or nasoenteral.ti,ab. or nasojejunal.ti,ab. or nasoduodenal.ti,ab. or postpyloric.ti,ab. or post-pyloric.ti,ab. or nasointestinal.ti,ab. or nasoenteric.ti,ab.

39. and/36-38

40. 35 and 39

For peer review only

CENTRAL

up to 30 September 2020.

1. electromagn*:ti,ab,kw

2. cortrak:ti,ab,kw

3. 1 or 2

4. [mh Endoscopy]

5. endoscop$:ti,ab,kw

6. 4 or 5

7. [mh “Enteral Nutrition”]

8. nasoenteral:ti,ab,kw

9. nasojejunal:ti,ab,kw

10. nasoduodenal:ti,ab,kw

11. postpyloric:ti,ab,kw

12. post-pyloric:ti,ab,kw

13. nasointestinal:ti,ab,kw

14. nasoenteric:ti,ab,kw

15. or/7-14

16. 3 and 6 and 15

For peer review only

BMJ Open

Electromagnetic-guided versus endoscopic placement of nasoenteral feeding tubes: protocol for a systematic review and meta-analysis

Journal:	*BMJ Open*
Manuscript ID	bmjopen-2020-044637.R2
Article Type:	Protocol
Date Submitted by the Author:	31-Jan-2021
Complete List of Authors:	Jin, Zheng; Affiliated Hangzhou First People's Hospital Zhejiang University School of Medicine<br>Wei, Yaping; Affiliated Hangzhou First People's Hospital Zhejiang University School of Medicine<br>Shen, Guofan; Affiliated Hangzhou First People's Hospital Zhejiang University School of Medicine<br>Zhang, Xiaofeng; Affiliated Hangzhou First People's Hospital Zhejiang University School of Medicine
**Primary Subject Heading**:	Emergency medicine
Secondary Subject Heading:	Gastroenterology and hepatology
Keywords:	Endoscopy < GASTROENTEROLOGY, Nutritional support < GASTROENTEROLOGY, Adult intensive & critical care < INTENSIVE & CRITICAL CARE

SCHOLARONE™
Manuscripts

I, the Submitting Author has the right to grant and does grant on behalf of all authors of the Work (as defined in the below author licence), an exclusive licence and/or a non-exclusive licence for contributions from authors who are: i) UK Crown employees; ii) where BMJ has agreed a CC-BY licence shall apply, and/or iii) in accordance with the terms applicable for US Federal Government officers or employees acting as part of their official duties; on a worldwide, perpetual, irrevocable, royalty-free basis to BMJ Publishing Group Ltd ("BMJ") its licensees and where the relevant Journal is co-owned by BMJ to the co-owners of the Journal, to publish the Work in this journal and any other BMJ products and to exploit all rights, as set out in our licence.

The Submitting Author accepts and understands that any supply made under these terms is made by BMJ to the Submitting Author unless you are acting as an employee on behalf of your employer or a postgraduate student of an affiliated institution which is paying any applicable article publishing charge ("APC") for Open Access articles. Where the Submitting Author wishes to make the Work available on an Open Access basis (and intends to pay the relevant APC), the terms of reuse of such Open Access shall be governed by a Creative Commons licence – details of these licences and which Creative Commons licence will apply to this Work are set out in our licence referred to above.

Other than as permitted in any relevant BMJ Author's Self Archiving Policies, I confirm this Work has not been accepted for publication elsewhere, is not being considered for publication elsewhere and does not duplicate material already published. I confirm all authors consent to publication of this Work and authorise the granting of this licence.

For peer review only

Title Page

Title

Electromagnetic-guided versus endoscopic placement of nasoenteral feeding tubes: protocol for a systematic review and meta-analysis

Authors

Zheng Jin MD*, Yaping Wei MD*, Guofan Shen MD*, Xiaofeng Zhang MD

Affiliated Hangzhou First People's Hospital, Zhejiang University School of Medicine, Hangzhou, China

*These authors have contributed equally to this work.

Correspondence to: Xiaofeng Zhang, MD, Department of Gastroenterology, Affiliated Hangzhou First People's Hospital, Zhejiang University School of Medicine, #261 Huansha Road, Hangzhou, 310006, Zhejiang Province, China.

Fax: +86-0571-56006872

E-mail address: gangdaoersan@163.com

For peer review only

ABSTRACT

Introduction: Current evidence supporting the utility of electromagnetic (EM)-guided method as the preferred technique for nasoenteral feeding tube placement is limited. We plan to provide a meta-analysis to compare the performance of EM-guided versus endoscopic placement.

Methods and analysis: Randomized controlled trials evaluating EM-guided vs. endoscopic placement will be searched in MEDLINE, EMBASE, and CENTRAL, from database inception to 30 September 2020. Data on study design, participant characteristics, intervention details and outcomes will be extracted. Primary outcomes to be assessed are complications. Secondary outcomes include procedure success rate, total procedure time, patient recommendation, length of hospital stay, and mortality. Study quality will be assessed using the Cochrane Risk of Bias Tool. Data will be combined with a random effect model. The results will be presented as a risk ratio for dichotomous data, and weighted mean difference for continuous data. Publication bias will be visualized using funnel plots. We will quantify the effect of potential effect modifiers by meta-regression if appropriate. The quality of evidence will be evaluated according to the GRADE framework.

For peer review only

For peer review only - http://bmjopen.bmj.com/site/about/guidelines.xhtml

Ethics and dissemination: This study will not use primary data, and therefore formal ethical approval is not required. The findings will be disseminated through peer-reviewed journals and committee conferences.

PROSPERO registration number: CRD42020172427

For peer review only

ARTICLE SUMMARY

Strengths and limitations of this study

1. This systematic review and meta-analysis will update the existing clinical evidence of placement of nasoenteral feeding tubes.

2. The thorough and transparent methodological approach undertaken will minimise the risk of possible biases. Quality of evidence will be assessed to provide confidence in the effect estimates.

3. Multiple methods for investigating heterogeneity can inform intervention and study design, but contingent on the number and size of available studies.

4. Common to any meta-analysis, some heterogeneity across and within studies may exist.

For peer review only - http://bmjopen.bmj.com/site/about/guidelines.xhtml

INTRODUCTION

Malnutrition and inability to eat are conditions often encountered in inpatients. For such patients, enteral nutrition is considered to be superior to parenteral nutrition since it reduces complications, improves patient outcome, and is more cheaper.[1, 2]

It is common practice to place a nasoenteral feeding tube for enteral nutrition in patients who are intolerant of intragastric nutrition.[3] Endoscopic technique is typically used, but requires patient transportation between wards, preprocedural fasting, and radiological confirmation of the tube’ s position. Since first reported by Phang et al. in 2006,[4] electromagnetic (EM)-guided technique has been increasingly used for nasoenteral feeding tube placement. With increasing availability and familiarity with this technique, several randomized controlled trials (RCTs)[5-8] have compared EM-guided versus endoscopic (ENDO) technique. EM and ENDO technique have been compared in only one systematic review until now.[9] That review by Gerritsen et al. involving only one relevant RCT (66 patients) concluded that the efficacy and safety of the two techniques did not differ significantly, but EM offered advantages in logistics. In view of several new RCTs published, we have planned to pool the evidence to further evaluate the performance of EM vs. ENDO.

METHODS

For peer review only - http://bmjopen.bmj.com/site/about/guidelines.xhtml

The review will be performed according to the recommendations specified in the Cochrane Handbook for Intervention Reviews.[10] The reporting of the review will follow the Preferred Reporting Items for Systematic Reviews and Meta-Analysis (PRISMA) statement.[11]

Criteria for considering studies for this review

Eligibility criteria are established in terms of the Population-Intervention-Comparison-Outcome-Study design framework. Studies will be selected according to the following criteria:

Participants

Included studies will involve adult patients with an indication for enteral nutrition via a nasoenteral feeding tube, as indicated by the treating physician and/or consulting dietitian. Both critically ill and non-critically ill patients will be included. Exclusion criteria will be high suspicion of stenosis of the upper gastrointestinal tract, esophageal varices, signs of upper gastrointestinal hemorrhage and pregnancy.

Interventions/comparison

The intervention comparisons are EM vs. ENDO. Studies with additional prokinetic agent administration will also be included.

Outcomes

For peer review only - http://bmjopen.bmj.com/site/about/guidelines.xhtml

Primary outcome: complications.

There are 6 secondary outcomes: procedure success rate (defined as the percentage of successful tube placement in the desired location as determined), total procedure time, patient recommendation, length of hospital stay, and mortality.

Study design

Only RCTs (including factorial, cross-over, sequential design, etc.) will be included. An abstract with sufficient data will also be considered. We will only include studies that are presented in English language due to constraints in translational resources.

Studies will be excluded if it meets at least one of the following criteria: (i) observational studies including case-control and cohort studies; (ii) single arm studies; (iii) case reports, reviews, editorials and letters to editor; (iv) duplicate studies, in vitro studies, or animal studies.

Search methods for identification of studies

Electronic searches

Two investigators (ZJ and YW) will independently search MEDLINE, EMBASE, and CENTRAL, for all entries through 30 September 2020 using the following search terms: "Cortrak", "electromagnetic", "endoscopic", "nasoenteral, or post-pyloric", and "tube(s), feeding, or nutrition". The search strategies (Online supplementary appendix I) will be

For peer review only - http://bmjopen.bmj.com/site/about/guidelines.xhtml

For peer review only

decided on after a discussion among all reviewers. We will assess eligibility of the retrieved articles by title and abstract using predetermined inclusion criteria. If this information is insufficient for eligibility assessment, we will review the full article. If any up-to-date evidence is published during the review period, we will evaluate the eligibility of each study and consider its addition to the analysis. To further increase the robustness of the literature search, a manual recursive search of the Web of Science for a list of studies that cite the included studies and studies that the included studies cited, will be carried out to identify other potentially relevant articles.

Data collection and analysis

Selection of studies

Decisions about study inclusion and exclusion will be made independently by two investigators (ZJ and YW). Disagreements will be resolved by consensus after a mutual discussion. The details of the study selection procedure are shown in a PRISMA flow chart. (Figure 1)

Data extraction and management

Two investigators (YW and GS) will independently extract the appropriate data onto Standardised extraction forms. The following data will be extracted from included trials: author, year of publication, country of origin, number of centers, participating operators, patient demographics, indications for enteral nutrition, and study outcomes. When

For peer review only - http://bmjopen.bmj.com/site/about/guidelines.xhtml

necessary data are not included in the published papers, the first or corresponding authors will be contacted for additional information. If there is no reply, we will analyse only the available data.

Assessment of risk of bias in included studies

We will assign two independent investigators (YW and GS) to appraise methodological quality of the included trials with the Cochrane Collaboration' s tool for assessing risk of bias.[12] The tool appraises existence of selection bias by assessing methods of randomization and allocation concealment, performance and detection of biases by checking blinding of personnel and outcome assessment, and attrition and reporting bias by evaluating incomplete and selective data reporting. Each of the item is assigned a judgment of high, low, or unclear risk.

Data synthesis

Risk ratios (RRs) will be calculated for categorical variables. Weighted mean differences (WMDs) will be calculated for continuous variables. Medians will be used if means are not available and standard deviations (SDs) will be calculated or imputed when possible.[13] Owning to the assumption of inherently various study scenarios and study populations, a random effects model for all analyses will be assumed. Heterogeneity among studies will be assessed by calculating the I^2 statistics whereby I^2<25% indicates no heterogeneity, 25%≤ I^2 <50% indicates mild heterogeneity, 50%≤ I^2 <75% indicates

For peer review only - http://bmjopen.bmj.com/site/about/guidelines.xhtml

moderate heterogeneity and I^2 ≥75% indicates strong heterogeneity.[14] We had planned that if sufficient studies (≥10) are included in the analysis of primary outcomes, we would construct funnel plots to evaluate publication bias.[15] Egger test for low sample size with a significance level of 0.05, and Begg and Mazumdar test for high sample volume at a significance level of 0.1.[16] All statistical analyses will be performed using Review Manager 5.4.1 (The Cochrane Collaboration, The Nordic Cochrane Centre, Copenhagen, Denmark).

Meta-regression analysis

A random-effects meta-regression analysis will be conducted to elucidate the impact of core set covariates (mean age, the proportion of males, sample size,research year and risk of bias) on treatment effects.

Subgroup analyses

In the case of possible strong heterogeneity, we will explore the possible sources using subgroup analyses. It is reported that a higher body mass index (BMI) were associated with a successful placement for EM.[5, 7] So, subgroup analyses will be carried out based on geographical location, study setting, the BMI level of patients with the validated method (ENDO group), prior altered upper gastrointestinal anatomy (with vs. without), patient population (critically ill vs. non-critically ill patients). For those subgroups with only 1 study included, subgroup analyses will not be performed.

For peer review only - http://bmjopen.bmj.com/site/about/guidelines.xhtml

Sensitivity analysis

A sensitivity analysis will be conducted to determine whether our results are robust. We will exclude the studies with high risk for bias from the summary analysis and analyse them again to assess the impact of these studies on the results.

Summary of evidence

We will summarise the quality of evidence using the GRADE (Grading of Recommendations Assessment, Development and Evaluation) approach and present ‘Summary of findings’ tables. Outcomes will be divided into critical (complications, procedure success rate, length of hospital stay, and mortality), and important (total procedure time, and patient recommendation) outcomes.

Patient and public involvement

Because the collected data within this systematic review and meta-analysis originates from previously published studies, patients and the general public were not involved in the development of the research question or choice of outcome measures that we wanted to assess.

DISCUSSION

Conventional methods for the placement of nasoenteral feeding tubes include blind, fluoroscopic, and endoscopic methods.[17, 18] The gold standard for the placement of

For peer review only - http://bmjopen.bmj.com/site/about/guidelines.xhtml

nasoenteral feeding tube is the endoscopic technique, which has success rates above 90%.[19, 20] Bed-side EM-guided tube placement can be performed in recent years. This has several potential advantages compared with endoscopic placement because only one trained nurse and less equipment is needed.[21, 22] But current evidence supporting the utility of EM-guided method as the preferred technique is limited. We therefore propose an updated meta-analysis to pool the evidence to further evaluate the performance of EM vs. ENDO. The results of this study will influence decision making for the patients who are malnutrition or inability to eat, assist in future guideline development and guide future research endeavors.

ETHICS AND DISSEMINATION

Ethics approval and patient written informed consent will not be required because all analyses in the present study will be performed based on data from published studies. We will disseminate the findings of our work through conference presentations and a peer-reviewed publication.

AUTHOR STATEMENT

Author Contributions

ZJ and XZ conceived and designed this study. ZJ and YW searched and selected the studies. YW and GS extracted the essential information. YW and GS assessed the risk of

For peer review only - http://bmjopen.bmj.com/site/about/guidelines.xhtml

bias. GS and ZJ performed the statistical analyses. ZJ and YW interpreted the pooled results. ZJ, YW and GS drafted the manuscript. All authors approved the manuscript to be considered for publication.

Competing interests: None.

Funding: None.

For peer review only

References:

1. Doig GS, Heighes PT, Simpson F, Sweetman EA, Davies AR. Early enteral nutrition, provided within 24 h of injury or intensive care unit admission, significantly reduces mortality in critically ill patients: a meta-analysis of randomised controlled trials. *Intensive Care Med*. 2009; **35**: 2018-27.

2. Jones NE, Heyland DK. Implementing nutrition guidelines in the critical care setting: a worthwhile and achievable goal? *JAMA*. 2008; **300**: 2798-9.

3. Hsu CW, Sun SF, Lin SL*, et al.*. Duodenal versus gastric feeding in medical intensive care unit patients: a prospective, randomized, clinical study. *CRIT CARE MED*. 2009; **37**: 1866-72.

4. Phang J, Marsh W, Prager R. Feeding Tube Placement with the Aid of a New Electromagnetic Transmitter. *JPEN-PARENTER ENTER*. 2006; **30**: S82.

5. Gao X, Zhang L, Zhao J*, et al.*. Bedside electromagnetic-guided placement of nasoenteral feeding tubes among critically Ill patients: A single-centre randomized controlled trial. *J CRIT CARE*. 2018; **48**: 216-21.

For peer review only - http://bmjopen.bmj.com/site/about/guidelines.xhtml

6. Gerritsen A, de Rooij T, Dijkgraaf MG, *et al.*. Electromagnetic-Guided Bedside Placement of Nasoenteral Feeding Tubes by Nurses Is Non-Inferior to Endoscopic Placement by Gastroenterologists: A Multicenter Randomized Controlled Trial. *AM J GASTROENTEROL*. 2016; **111**: 1123-32.

7. Holzinger U, Brunner R, Miehsler W, *et al.*. Jejunal tube placement in critically ill patients: A prospective, randomized trial comparing the endoscopic technique with the electromagnetically visualized method. *CRIT CARE MED*. 2011; **39**: 73-7.

8. Kappelle W, Walter D, Stadhouders PH, *et al.*. Electromagnetic-guided placement of nasoduodenal feeding tubes versus endoscopic placement: a randomized, multicenter trial. *GASTROINTEST ENDOSC*. 2018; **87**: 110-8.

9. Gerritsen A, van der Poel MJ, de Rooij T, *et al.*. Systematic review on bedside electromagnetic-guided, endoscopic, and fluoroscopic placement of nasoenteral feeding tubes. *GASTROINTEST ENDOSC*. 2015; **81**: 836-47.

10. Higgins JPT, Thomas J, Chandler J, *et al.*. Cochrane Handbook for Systematic Reviews of Interventions version 6.1., 2020.

11. Moher D, Liberati A, Tetzlaff J, Altman DG. Preferred reporting items for systematic reviews and meta-analyses: the PRISMA statement. *BMJ*. 2009; **339**: b2535.

12. Higgins JP, Altman DG, Gotzsche PC, *et al.*. The Cochrane Collaboration's tool for assessing risk of bias in randomised trials. *BMJ*. 2011; **343**: d5928.

13. Hozo SP, Djulbegovic B, Hozo I. Estimating the mean and variance from the median, range, and the size of a sample. *BMC MED RES METHODOL*. 2005; **5**: 13.

14. Higgins JP, Thompson SG. Quantifying heterogeneity in a meta-analysis. *STAT MED*. 2002; **21**: 1539-58.

15. Higgins JP, Green S. Cochrane Handbook for Systematic Reviews of Interventions., 2011.

16. Egger M, Davey SG, Schneider M, Minder C. Bias in meta-analysis detected by a simple, graphical test. *BMJ*. 1997; **315**: 629-34.

17. Hillard AE, Waddell JJ, Metzler MH, McAlpin D. Fluoroscopically guided nasoenteric feeding tube placement versus bedside placement. *SOUTH MED J*. 1995; **88**: 425-8.

18. Scott R, Bowling TE. Enteral tube feeding in adults. *J R Coll Physicians Edinb*. 2015; **45**: 49-54.

For peer review only - http://bmjopen.bmj.com/site/about/guidelines.xhtml

19. Byrne KR, Fang JC. Endoscopic placement of enteral feeding catheters. *Curr Opin Gastroenterol*. 2006; **22**: 546-50.

20. Wiggins TF, DeLegge MH. Evaluation of a new technique for endoscopic nasojejunal feeding-tube placement. *GASTROINTEST ENDOSC*. 2006; **63**: 590-5.

21. Kline AM, Sorce L, Sullivan C, Weishaar J, Steinhorn DM. Use of a Noninvasive Electromagnetic Device to Place Transpyloric Feeding Tubes in Critically Ill Children. *AM J CRIT CARE*. 2011; **20**: 453-60.

22. Arjaans W, Ouwehand M, Bouma G, van der Meulen T, de van der Schueren M. Cortrak((R)) duodenal tube placements: A solution for more patients? A preliminary survey to the introduction of electromagnetic-guided placement of naso-duodenal feeding tubes. *Clin Nutr ESPEN*. 2019; **29**: 133-6.

For peer review only

Figure 1. Flow diagram of the study selection process.

Online supplementary appendix 1: Search strategies

For peer review only

For peer review only - http://bmjopen.bmj.com/site/about/guidelines.xhtml

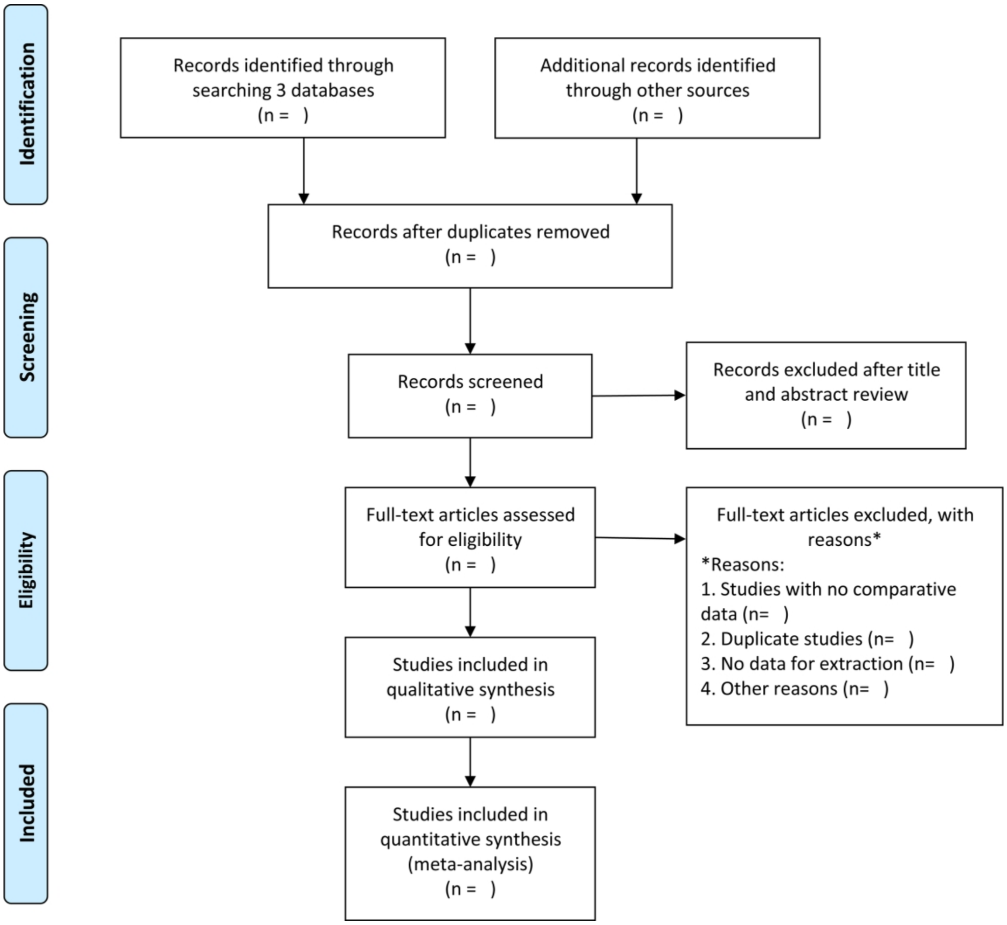

Flow diagram of the study selection process.

190x174mm (300 x 300 DPI)

Online supplementary appendix 1. Search strategies

MEDLINE (PubMed format)

up to 30 September 2020.

1. electromagn* [tiab] OR cortrak [tiab]
2. Endoscopy [mesh] OR endoscop* [tiab]
3. Enteral Nutrition [mesh] OR nasoenteral [tiab] OR nasojejunal [tiab] OR nasoduodenal [tiab] OR postpyloric [tiab] OR post-pyloric [tiab] OR nasointestinal [tiab] OR nasoenteric [tiab]
4. randomized controlled trial [pt] OR controlled clinical trial [pt] OR randomized [tiab] OR placebo [tiab] OR clinical trials as topic [mesh: noexp] OR randomly [tiab] OR trial [ti]
5. animals [mesh] NOT humans [mesh]
6. 4 NOT 5
7. 1 AND 2 AND 3 AND 6

EMBASE (Ovid format)

up to 30 September 2020.

1. Randomized controlled trial/
2. Controlled clinical study/
3. random$.ti,ab.
4. randomization/
5. intermethod comparison/

For peer review only

6. placebo.ti,ab.

7. (compare or compared or comparison).ti.

8. ((evaluated or evaluate or evaluating or assessed or assess) and (compare or compared or comparing or comparison)).ab.

9. (open adj label).ti,ab.

10. ((double or single or doubly or singly) adj (blind or blinded or blindly)).ti,ab.

11. double blind procedure/

12. parallel group$1.ti,ab.

13. (crossover or cross over).ti,ab.

14. ((assign$ or match or matched or allocation) adj5 (alternate or group$1 or intervention$1 or patient$1 or subject$1 or participant$1)).ti,ab.

15. (assigned or allocated).ti,ab.

16. (controlled adj7 (study or design or trial)).ti,ab.

17. (volunteer or volunteers).ti,ab.

18. human experiment/

19. trial.ti.

20. or/1-19

21. random$ adj sampl$ adj7 ("cross section$" or questionnaire$1 or survey$ or database$1)).ti,ab. not (comparative study/ or controlled study/ or randomi?ed controlled.ti,ab. or randomly assigned.ti,ab.)

22. Cross-sectional study/ not (randomized controlled trial/ or controlled clinical study/ or controlled study/ or randomi?ed controlled.ti,ab. or control group$1.ti,ab.)

For peer review only

23. (((case adj control$) and random$) not randomi?ed controlled).ti,ab.

24. (Systematic review not (trial or study)).ti.

25. (nonrandom$ not random$).ti,ab.

26. “Random field$”.ti,ab.

27. (random cluster adj3 sampl$).ti,ab.

28. (review.ab. and review.pt.) not trial.ti.

29. “we searched”.ab. and (review.ti. or review.pt.)

30. “update review”.ab.

31. (databases adj4 searched).ab.

32. (rat or rats or mouse or mice or swine or porcine or murine or sheep or lambs or pigs or piglets or rabbit or rabbits or cat or cats or dog or dogs or cattle or bovine or monkey or monkeys or trout or marmoset$1).ti. and animal experiment/

33. Animal experiment/ not (human experiment/ or human/)

34. or/21-33

35. 20 not 34

36. electromagn$.ti,ab. or cortrak.ti,ab.

37. Endoscopy/exp or endoscop$.ti,ab.

38. “Enteral Nutrition”/exp or nasoenteral.ti,ab. or nasojejunal.ti,ab. or nasoduodenal.ti,ab. or postpyloric.ti,ab. or post-pyloric.ti,ab. or nasointestinal.ti,ab. or nasoenteric.ti,ab.

39. and/36-38

40. 35 and 39

For peer review only

CENTRAL

up to 30 September 2020.

1. electromagn*:ti,ab,kw

2. cortrak:ti,ab,kw

3. 1 or 2

4. [mh Endoscopy]

5. endoscop$:ti,ab,kw

6. 4 or 5

7. [mh "Enteral Nutrition"]

8. nasoenteral:ti,ab,kw

9. nasojejunal:ti,ab,kw

10. nasoduodenal:ti,ab,kw

11. postpyloric:ti,ab,kw

12. post-pyloric:ti,ab,kw

13. nasointestinal:ti,ab,kw

14. nasoenteric:ti,ab,kw

15. or/7-14

16. 3 and 6 and 15

For peer review only
